# Supplementary material for: Uncovering Novel Features of the Pc Locus in Horn Development from Gene-Edited Holstein Cattle by RNA-Sequencing Analysis
Source: Int J Mol Sci. 2022 Oct 11;23(20):12060. doi: 10.3390/ijms232012060 (PMC9603690; doi:10.3390/ijms232012060)
Supplement: Supplementary file 1 [file ijms-23-12060-s001.zip › Table S3.pdf]

Table S3. Primer sequences for PCR

Primer Name	Primer Sequence	annealing temperature	PCR product size
btHP-1748-F	5’-GGGCAAGTTGCTCAGCTGTTTTTG-3’	58℃	1546 bp
btHP-1594-R	5’-TGAATCCTGCTAAACCATGCGGA-3’		
NEW-F	5’- CCATGAGAATTAGAGTGGGATGC-3’	58℃	1318 bp
NEW-R	5’- CATGGTTCACTCATAGTGACTCC-3’		

Sequence of Pc locus

CTGTGAAATGAAGAGTACGTGGTACCAACTACTTTCTGAGCTCACGCACAGCTGGACG

TCTGCGCCTTTCTTGTTATACTGCAGATGAAAACATTTTATCAGATGTTTGCCTAAGTAT

GGATTACATTTAAGATACATATTTTTCTTTCTTGTCTGAAAGTCTTTGTAGTGAGAGCAG

GCTGGAATTATGTCTGGGGTGAGATAGTTTTCTT
