# Supplementary material for: Uncovering Novel Features of the Pc Locus in Horn Development from Gene-Edited Holstein Cattle by RNA-Sequencing Analysis
Source: Int J Mol Sci. 2022 Oct 11;23(20):12060. doi: 10.3390/ijms232012060 (PMC9603690; doi:10.3390/ijms232012060)
Supplement: Supplementary file 1 [file ijms-23-12060-s001.zip › Table S4.pdf]

Table S4. Primer sequences for RT-qPCR

Gene ID	Gene Primer Name	Primer Sequence
507053	RXFP2-F	5'- AGAACCCCACAATCCAGATG-3'
	RXFP2-R	5'- TGACCCTGGAGAAGTTCCTG-3'
782170	TWIST1-F	5'- TGTGAGTCAGTTTGATCCCAAT-3'
	TWIST1-R	5'- GGCATCATTATGGACTTTCTCC-3'
282637	CDH1-F	5'- GGCTGGACCGTGAGAGTTTT-3'
	CDH1-R	5'- GCAGTTGTGCTCAAGCCTTC-3'
282637	ITGB3-F	5'- TGACATCCTGGTGGTCTTGC-3'
	ITGB3-R	5'- AGTGGGTTGTTGGCAGTGTC-3'
281592	ACTA1-F	5'- GAAGTCTCGCTTCCTTCCCA-3'
	ACTA1-R	5'- GGTATCTGGTTTCTGGGCGG-3'
536607	ACTN2-F	5'- GAGGCTAGCGAGTGAGCTTT-3'
	ACTN2-R	5'- CTTCTCCTGCACCTTGGGAG-3'
539375	ACTN3-F	5'- CACTTTGACCGGAAGCGGAA-3'
	ACTN3-R	5'- TCATGATGCGAGCGAACTCC-3'
538107	ASXL3-F	5'- CCACGTCATCAGCAGAACCA-3'
	ASXL3-R	5'- TTTTTCTAGTGCCAGGCGGG-3'
353115	SOX10-F	5'- GCAAGCTCTGGAGGCTGCT-3'
	SOX10-R	5'- GGTGGGCGCTCTTGTAGTG-3'
281181	GAPDH-F	5'- GGGTCATCATCTCTGCACCT-3'
	GAPDH-R	5'- GGTCATAAGTCCCTCCACGA-3'
